# Beyond Euthyroid Sick Syndrome: Pre-Admission Levothyroxine Use and Thyroid Hormone–Outcome Associations in Hospitalized Patients

**DOI:** 10.3390/jcm15218382

**Published:** 2026-10-28

**Authors:** Mohammad Sheikh-Ahmad, Ilana Rosenblatt, Hadas Rabani, Clara Henig, Balsam Dakwar, Ekaterina Yovanovich, Emad Khoury, Leonard Saiegh

**Affiliations:** 1 Institute of Endocrinology, Bnai Zion Medical Center, 47 Eliyahu Golomb Avenue, Haifa 3339419, Israel; rosenblatt.ilana@gmail.com (I.R.); hadas.rabani@b-zion.org.il (H.R.); balsam.dakwar@b-zion.org.il (B.D.); katya.rogachevsky@b-zion.org.il (E.Y.); emad.khoury@b-zion.org.il (E.K.); leonard.saiegh@b-zion.org.il (L.S.); 2 The Ruth and Bruce Rappaport Faculty of Medicine, Technion, Israel Institute of Technology, 1 Efron Street, Haifa 3339101, Israel; 3 Clinical Laboratories Division, Bnai Zion Medical Center, Haifa 3339419, Israel; clara.henig@b-zion.org.il

**Keywords:** non-thyroidal illness syndrome, levothyroxine, hypothyroidism, free triiodothyronine, free thyroxine, hospitalization, propensity-score matching, retrospective cohort study

## Abstract

**Background/Objectives:** Pre-admission levothyroxine use may influence the interpretation of thyroid tests beyond the conventional framework of non-thyroidal illness syndrome (NTIS). We examined whether thyroid hormone–outcome associations differed by treatment status. **Methods:** This retrospective study included medical inpatients with thyroid-stimulating hormone (TSH) < 5 mIU/L during 2006–2024. Propensity-score matching used age, sex, and 16 comorbidity indicators. Complete-case negative-binomial and logistic models examined hospitalization length and in-hospital mortality, with treatment-by-hormone interactions. **Results:** Among 14,332 matched patients, 3717 had complete data (1986 users; 1731 nonusers). Higher free triiodothyronine (FT3) in nonusers was associated with shorter hospitalization (incidence–rate ratio [IRR] 0.76, 95% confidence interval [CI] 0.73–0.80) and lower mortality odds (odds ratio [OR] 0.47, 95% CI 0.35–0.62). Free thyroxine (FT4) associations differed by levothyroxine use for both hospitalization length (interaction IRR 0.95, 95% CI 0.94–0.97) and mortality (interaction OR 0.83, 95% CI 0.77–0.90; both *p* < 0.001). At mean hormone concentrations, levothyroxine use was associated with shorter hospitalization (IRR 0.87, 95% CI 0.81–0.94) and lower mortality odds (OR 0.53, 95% CI 0.35–0.82). Crude mortality was 4.6% versus 5.0%, respectively; the conditional estimates represent a different comparison. **Conclusions:** FT4–outcome associations differed by pre-admission levothyroxine use, extending the established low-T3 prognostic framework to treatment-specific hormone patterns. These findings support considering treatment history when interpreting inpatient thyroid tests. Selective testing and residual confounding limit causal interpretation.

## 1. Introduction

Non-thyroidal illness syndrome (NTIS), also termed euthyroid sick syndrome or low-T3 syndrome, describes the changes in thyroid-hormone physiology accompanying systemic illness. Reduced triiodothyronine (T3), variable thyroxine (T4), and an absent or blunted thyroid-stimulating hormone (TSH) response reflect changes in peripheral metabolism and hypothalamic–pituitary signaling [1,2]. NTIS provides an important framework for interpreting inpatient thyroid tests, including in patients with established thyroid disease [3].

NTIS, primary hypothyroidism, and the biochemical effects of levothyroxine are related but distinct clinical contexts. Intrinsic thyroid disease and illness-associated hormone changes can coexist, and a single inpatient TSH value cannot establish pre-illness thyroid status. Levothyroxine supplies exogenous T4, while circulating T3 reflects both thyroidal secretion and peripheral conversion; treatment can therefore alter the relationship between FT4 and FT3 even when TSH is within the reference range [1,4,5]. These distinctions are particularly relevant when comparing treated and untreated medical inpatients.

Whether NTIS represents an adaptive response that limits tissue catabolism or a maladaptive process contributing to morbidity remains a long-standing debate [6,7,8,9]. Acute down-regulation of the hypothalamic–pituitary–thyroid (HPT) axis is often interpreted as an adaptive response that limits energy expenditure during physiological stress, whereas persistent low-T3 states during protracted illness may contribute to impaired recovery and, in some patients, transient central hypothyroidism [2,10]. Low T3 has repeatedly been identified as an independent predictor of adverse outcomes in diverse populations, including critically ill adults [11,12], patients with ischemic heart failure [13,14,15], and hospitalized patients with COVID-19 [16].

Despite the association of low T3 with adverse outcomes, trials of thyroid-hormone administration during acute illness have not established a consistent clinical benefit [3,17,18,19]. American Thyroid Association guidance recommends against levothyroxine specifically for NTIS in critically ill hospitalized patients [20]. This question differs from interpreting hormone concentrations in patients already receiving replacement for an established thyroid disorder.

Pre-admission levothyroxine use introduces an additional source of variation in inpatient hormone concentrations. In users, FT4 may reflect replacement dose, absorption, dose-to-sampling time, and residual thyroid function, superimposed on the metabolic response to illness. In nonusers, the same FT4 concentration arises without exogenous T4. Whether hormone–outcome associations differ between these clinical settings is therefore a separate question from whether low T3 accompanies a poor prognosis.

We examined associations of inpatient TSH, FT3, and FT4 with hospitalization length and in-hospital mortality, and tested whether these associations differed by documented pre-admission levothyroxine use. The principal contribution is the treatment-by-hormone comparison, particularly the FT4–outcome association, within the broader NTIS framework. Death within 31 days after discharge was assessed as a secondary, unadjusted outcome. The cohort was defined by testing and exposure criteria rather than an individual adjudication of NTIS.

## 2. Materials and Methods

### 2.1. Study Design and Setting

We conducted a single-center retrospective cohort study at Bnai Zion Medical Center, Haifa, Israel, using institutional electronic medical records. Adults aged 18–90 years admitted to three internal-medicine departments between January 2006 and December 2024 were eligible if TSH was measured during hospitalization. Testing was routine in one department and ordered at the treating physician’s discretion in the other two. For patients with repeated eligible admissions, only the first qualifying admission was retained. For repeated thyroid measurements, the first inpatient result was used.

Patients with the thyroid-disease codes listed in Table S1, principally hyperthyroidism or thyroiditis, and patients whose levothyroxine was initiated or discontinued during the index admission were excluded. The analytic cohort was restricted to TSH < 5 mIU/L, without a lower cutoff. This was an eligibility criterion, not a definition of euthyroidism or NTIS. The detailed code sets define operational indicators rather than comprehensive clinical diagnoses.

The exposure was documented levothyroxine use at admission, verified from hospital pharmacy records and community-pharmacy medication lists. Nonusers had no documented use at admission. Treatment duration, dose, indication, adherence, and outpatient biochemical control were not characterized; accordingly, the exposure is termed pre-admission use rather than verified long-term, stable replacement.

The primary outcomes were total length of hospitalization in days and in-hospital mortality. Total length of stay includes time before the thyroid sample was obtained. The secondary outcome was death within 31 days after discharge, with discharge as the time origin and only patients discharged alive in the denominator. Mortality was ascertained from hospital records and post-hospitalization follow-up documented in the electronic patient record.

FT3 and FT4 were ordered at the treating physician’s discretion in all three departments. FT3 became available on 31 July 2012; thus, complete-case models requiring FT3 concern testing from that date onward. Admission-to-sampling intervals, synchronization of the different laboratory measurements, and serial hormone trajectories were not analyzed. Sampling was not timed relative to levothyroxine administration.

White blood cell count (WBC) and C-reactive protein (CRP) were included as inflammatory covariates. The propensity score used age, sex, and 16 comorbidity indicators: type 2 or unspecified diabetes, type 1 diabetes, hypertension, ischemic heart disease, hyperlipidemia, non-alcoholic liver disease, chronic liver disease/cirrhosis, heart failure, renal disease, anemia, obesity/overweight, chronic respiratory failure, chronic obstructive asthma, sleep apnea, cerebrovascular disease, and a composite of other chronic conditions. The indicator labels and ICD-9-CM definitions are specified in Table S1; codes were assigned before or during admission.

### 2.2. Assays

Serum samples were obtained as part of routine clinical care, collected in accordance with standard pre-analytical guidelines, and analyzed using automated immunoassay platforms that evolved over time. All measurements were performed on serum after centrifugation and according to standardized laboratory operating procedures.

Between 1 January 2006 and 31 May 2007, TSH and free thyroxine (FT4) were measured using the AxSYM analyzer (Abbott Laboratories, Abbott Park, IL, USA). Both assays were performed using microparticle enzyme immunoassay technology. The TSH assay had a measuring range of 0.006–40 μIU/mL, with an analytical sensitivity of 0.006 μIU/mL and an inter-assay coefficient of variation (CV) < 15%. FT4 was measured over a range of 5.15–64.35 pmol/L, with an analytical sensitivity of 5.15 pmol/L and inter-assay CV < 10%. Between 1 June 2007 and 28 February 2008, TSH and FT4 were measured using the ARCHITECT i2000 analyzer (Abbott Laboratories, Abbott Park, IL, USA). Both assays were performed using automated two-step chemiluminescent microparticle immunoassay technology. The TSH assay had a measuring range of 0.0038–100.0 μIU/mL, with an analytical sensitivity of 0.0025 μIU/mL and inter-assay coefficient of variation (CV) < 10%. FT4 was measured over a range of 5.15–64.35 pmol/L, with an analytical sensitivity of 5.15 pmol/L and inter-assay CV < 10%. From 1 March 2008, to 31 July 2012, TSH and FT4 measurements were performed using the Immulite^®^ 2000 system (Siemens Healthineers, Erlangen, Germany). TSH was determined using a solid-phase, two-site chemiluminescent immunometric assay, with a measuring range of 0.004–75 μIU/mL, functional sensitivity of 0.003 μIU/mL, and inter-assay CV < 10%. FT4 was measured using a solid-phase, enzyme-labeled chemiluminescent competitive immunoassay, with a measuring range of 3.9–77.2 pmol/L, sensitivity of 2.83 pmol/L, and inter-assay CV < 10%. From 1 August 2012, onward, TSH, FT3, and FT4 were analyzed using the Cobas^®^ Elecsys 411 platform (Roche Diagnostics GmbH, Mannheim, Germany), based on electrochemiluminescence immunoassay (ECLIA) technology. TSH was measured using a sandwich immunoassay with a measuring range of 0.005–100 μIU/mL, functional sensitivity of 0.005 μIU/mL, and inter-assay CV < 6%. FT3 and FT4 were determined using competitive ECLIA assays. The FT3 assay had a measuring range of 0.6–50 pmol/L, sensitivity of 0.6 pmol/L, and inter-assay CV < 12%, while FT4 was measured over a range of 0.5–100 pmol/L, with a sensitivity of 0.5 pmol/L and inter-assay CV < 7%.

Assays followed manufacturers’ instructions, internal quality control, and external quality assurance. The source population spans four platforms and three transitions. FT3 introduction on 31 July 2012 and the Cobas transition on 1 August 2012 delimit the transition to the later testing period. Consequently, the three-hormone models concern a much narrower assay era than the 2006–2024 parent cohort. Platform was not included as a model covariate, and no platform-restricted results are presented.

### 2.3. Statistical Analysis

Continuous variables are summarized as mean ± standard deviation (SD) and were compared using the Mann–Whitney U test. Categorical variables are presented as counts and percentages or proportions and were compared using χ^2^ tests. The post-discharge mortality comparison among discharge survivors used Pearson’s χ^2^ test without continuity correction. A logistic-regression propensity score for levothyroxine use included age, sex, and the 16 comorbidity indicators. Matching was 1:1 on the logit of the score using nearest neighbors, a caliper of 0.2 times its pooled SD, and no replacement; ties were broken by order of selection and pairs outside common support were discarded. Absolute standardized mean differences (SMDs) assessed covariate balance, with 0.10 as a descriptive threshold [21,22]. Complete-case SMDs were calculated from group means and SDs or exact binary counts; those based on rounded continuous summaries are approximate.

Hospitalization length was modeled using a negative-binomial distribution with a log link, and in-hospital mortality using logistic regression. Each model included 16 model terms excluding the intercept: sex, age, levothyroxine use, diabetes, hypertension, ischemic heart disease, heart failure, renal disease, WBC, CRP, TSH, FT3, FT4, and the three levothyroxine-by-hormone interactions. The covariates represent demographics, selected comorbidities, inflammation, and the hormone terms relevant to the study question. Continuous covariates were mean-centered.

With interactions present, a hormone main term describes the per-unit association in nonusers. An interaction is the ratio of the corresponding per-unit associations in users and nonusers. The levothyroxine main term compares users with nonusers at the mean-centered reference values of TSH, FT3, and FT4, conditional on the remaining covariates; it is not a population-average treatment comparison. Approximate within-user hormone IRRs or ORs were obtained by multiplying the rounded main and interaction estimates. These products are descriptive point estimates; separate confidence intervals were not calculated. Confidence intervals for the interaction terms are presented directly.

Regression analyses used patients with complete values for every included covariate. FT3 missingness included its absence from clinical testing before July 2012 and selective ordering thereafter. The regression analyses used complete cases selected after the original matching. Pair-specific estimates or matched-pair-adjusted uncertainty estimates are not presented. Missing values were not imputed.

Results are expressed as incidence-rate ratios (IRRs) for hospitalization length, interpreted as ratios of expected hospital days, and odds ratios (ORs) for in-hospital mortality, with 95% confidence intervals (CIs). Model comparisons used likelihood-ratio tests against intercept-only models and the Akaike information criterion (AIC). In-hospital mortality included 179 events for 16 model terms excluding the intercept (approximately 11.2 events per term). The secondary post-discharge outcome was compared without covariate adjustment among discharge survivors; no adjusted post-discharge model is presented.

*p*-values are two-sided and nominal, without multiplicity correction. The two primary models include six treatment-by-hormone tests, interpreted as exploratory association analyses. The aggregate post-discharge comparison assumes independent observations and does not account for retained matched pairs. The statistical analyses used R version 4.5.2 and IBM SPSS Statistics version 29. The survivor-based contingency-table calculation and summary-based SMD calculations used Python version 3.12.

## 3. Results

**Figure 1:**
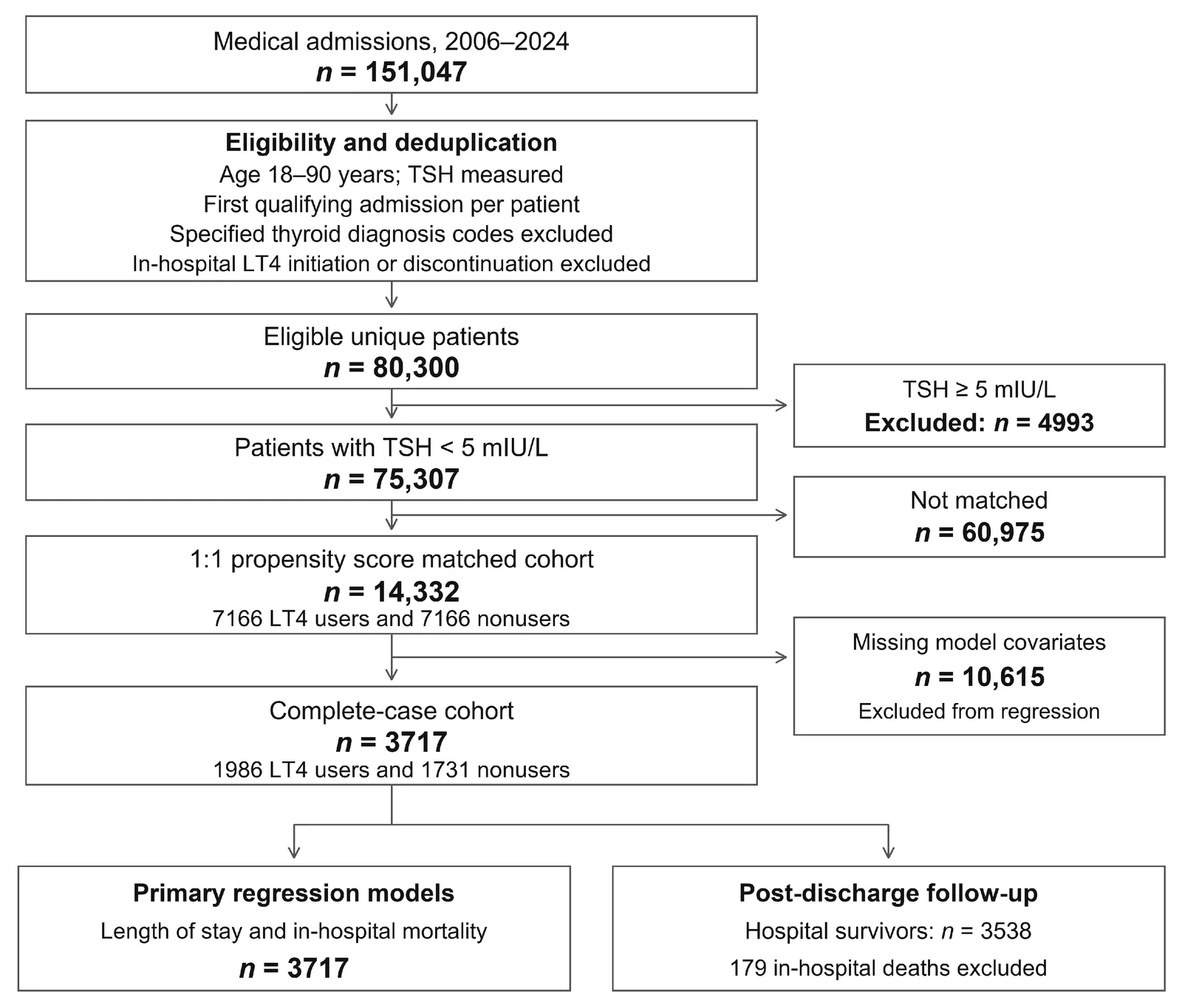
Cohort selection and outcome populations. Only the first qualifying admission per patient was retained. The counts at the principal selection stages distinguish admissions from unique patients. FT3 became available on 31 July 2012, limiting the complete-case models to the later testing period. The 179 patients who died in hospital were excluded from the post-discharge risk set. LT4 = levothyroxine.

Among 151,047 admissions, 80,300 unique patients met the initial eligibility criteria; exclusion of 4993 with TSH ≥ 5 mIU/L left 75,307 patients. Matching yielded 14,332 patients, with 7166 users and 7166 nonusers (Figure 1). The matching covariates were balanced in this parent cohort (Table 1), with all SMDs below 0.05. Mean age was 74.27 ± 12.89 years in users and 74.30 ± 12.94 years in nonusers; approximately 78% in each group were female.

Missingness in the matched cohort was approximately 74% for FT3, 57% for FT4, 1.3% for CRP, and 0.3% for WBC. The regression sample included 3717 complete cases (25.9% of matched patients): 1986 users and 1731 nonusers. It therefore retained neither equal treatment-group sizes nor the original 1:1 matched structure. All complete cases required a result from the FT3-available period.

Complete and incomplete cases differed in several characteristics (Table S2): hypertension was present in 41% versus 57%, ischemic heart disease in 12% versus 26%, and the renal-disease indicator in 14% versus 3%, respectively. These selection differences are distinct from the balance between users and nonusers within the complete-case sample.

In the complete-case sample, users had lower FT3 (2.73 ± 1.01 versus 3.20 ± 1.07 pmol/L) and higher FT4 (17.19 ± 5.38 versus 15.70 ± 3.72 pmol/L) than nonusers (both *p* < 0.001; Table 2). The corresponding absolute SMDs were 0.452 and 0.322. SMDs were 0.100 for CRP, 0.096 for heart failure, and 0.079 for renal disease, demonstrating the need to distinguish complete-case balance from balance in the matched parent cohort. Mean hospitalization length was 7.57 ± 15.06 versus 7.71 ± 13.09 days (*p* = 0.99). Crude in-hospital mortality was 92/1986 (4.6%) versus 87/1731 (5.0%; *p* = 0.58), respectively.

### 3.1. Length of Hospitalization

The negative binomial model for hospitalization length improved on the intercept-only model (likelihood-ratio χ^2^(16) = 295.3, *p* < 0.001; AIC = 22,752.7; Table 3).

Among nonusers, each 1 pmol/L higher FT3 was associated with shorter expected hospitalization (IRR 0.76, 95% CI 0.73–0.80; *p* < 0.001), whereas higher FT4 was associated with longer hospitalization (IRR 1.05, 95% CI 1.03–1.06; *p* < 0.001). At mean hormone concentrations and conditional on the other model covariates, levothyroxine use was associated with shorter expected hospitalization (IRR 0.87, 95% CI 0.81–0.94; *p* < 0.001). TSH was not associated with hospitalization length (*p* = 0.88).

**Figure 2:**
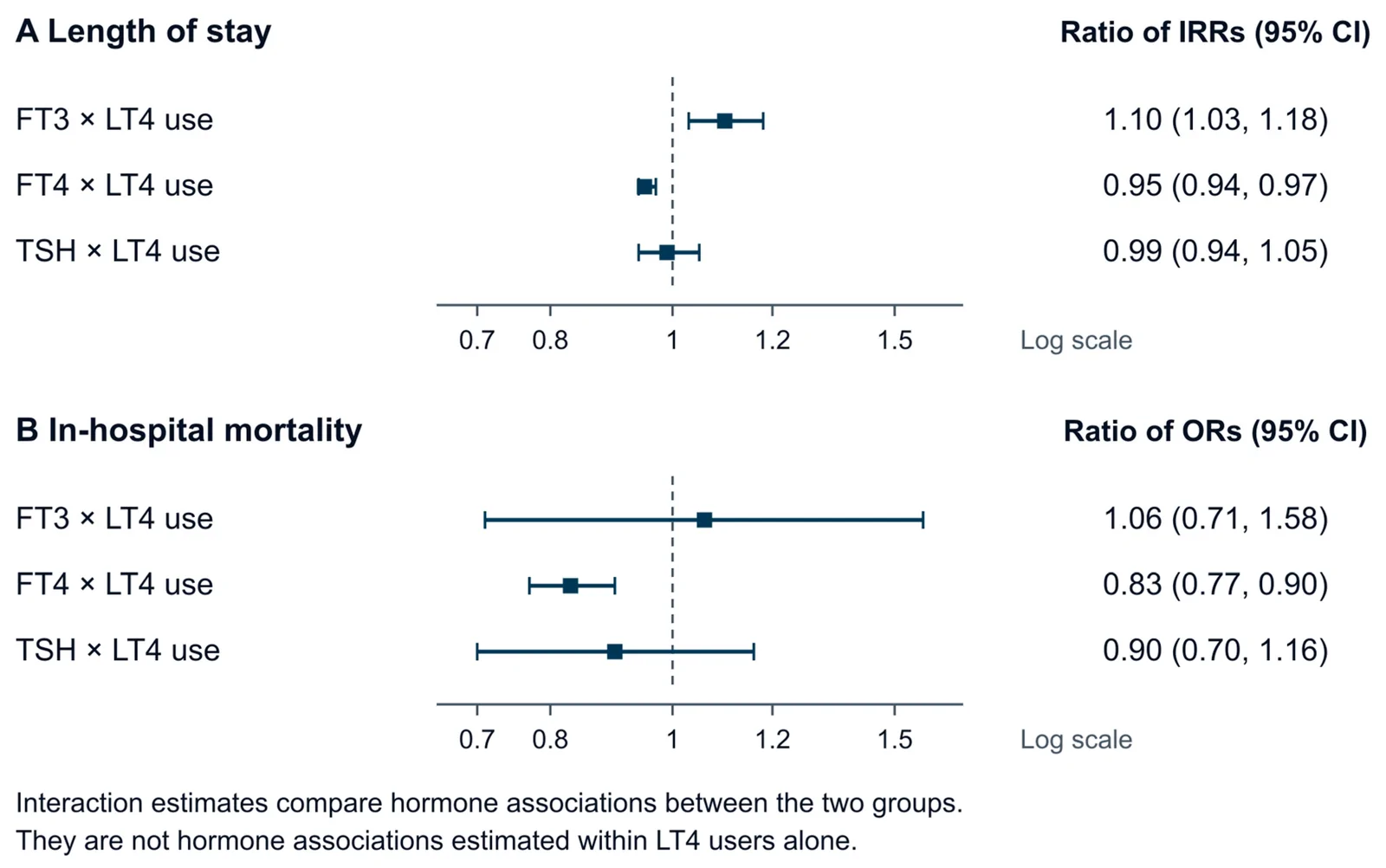
Thyroid hormone-by-levothyroxine interactions for hospitalization length (**A**) and in-hospital mortality (**B**). Points and bars show interaction estimates and 95% CIs. Each estimate is the ratio of the per-unit hormone association in users to that in nonusers: ratios of IRRs in A and ratios of ORs in B. The hormone units are 1 pmol/L for FT3 and FT4 and 1 mIU/L for TSH. The reference line at 1 indicates equal per-unit associations in the two groups. These estimates compare hormone associations between groups; they are not within-user associations or treatment effects.

The FT3-by-treatment interaction IRR was 1.10 (95% CI 1.03–1.18; *p* = 0.005), indicating a less pronounced inverse FT3 association in users. The FT4-by-treatment interaction IRR was 0.95 (95% CI 0.94–0.97; *p* < 0.001), indicating attenuation of the positive FT4 association. The approximate within-user point IRRs were 0.84 for FT3 and 1.00 for FT4, calculated from products of the rounded coefficients. Figure 2 displays the interaction estimates and their CIs.

### 3.2. In-Hospital Mortality

There were 179 in-hospital deaths among 3717 complete cases (4.8%). The logistic model improved on the intercept-only model (likelihood-ratio χ^2^(16) = 214.6, *p* < 0.001; AIC = 1254.6; Table 4).

Among nonusers, higher FT3 was associated with lower odds of in-hospital death (OR 0.47 per 1 pmol/L, 95% CI 0.35–0.62; *p* < 0.001). The FT4 point estimate was positive (OR 1.06, 95% CI 1.00–1.13; *p* = 0.057). The FT4-by-treatment interaction was significant (OR 0.83, 95% CI 0.77–0.90; *p* < 0.001), with an approximate within-user FT4 point OR of 0.88. The interaction indicates a difference in the modeled FT4 associations between groups; a separate confidence interval for the within-user association is not presented. At mean hormone concentrations, levothyroxine use was associated with lower mortality odds (OR 0.53, 95% CI 0.35–0.82; *p* = 0.004). The FT3-by-treatment interaction was not significant for mortality (OR 1.06, 95% CI 0.71–1.58; *p* = 0.783).

### 3.3. Mortality Within 31 Days After Discharge

Among 3538 patients discharged alive, 128 died within 31 days (3.6%): 65/1894 users (3.4%) and 63/1644 nonusers (3.8%). The unadjusted comparison showed no statistically significant difference (Pearson χ^2^(1) = 0.404; *p* = 0.525). These results describe the discharge-survivor population and are not covariate-adjusted estimates.

The treatment-by-hormone estimates from both primary models are displayed in Figure 2. FT4 interactions were evident for hospitalization length and in-hospital mortality; the FT3 interaction was evident for hospitalization length only.

## 4. Discussion

In this retrospective cohort of medical inpatients selected from a propensity-score-matched population, we identified three findings with potential clinical relevance. First, higher FT3 was associated with shorter hospitalization and lower in-hospital mortality odds among nonusers, the reference group in the interaction models; the inverse association with hospitalization length was less pronounced in levothyroxine users. Second, at mean thyroid-hormone concentrations, pre-admission levothyroxine use was associated with 13% shorter expected hospitalization and 47% lower odds of in-hospital death. Third, FT4-by-treatment interactions were present for both inpatient outcomes. This treatment-specific FT4 finding extends the established low-T3 prognostic framework by identifying treatment history as a relevant context for interpreting the components of an NTIS-like biochemical pattern.

The association of low FT3 with adverse inpatient outcomes is consistent with a substantial body of evidence from critical illness, cardiovascular disease, and COVID-19 [11,12,13,14,15,16]. Vidart et al. reported a pooled mortality OR of 2.21 (95% CI 1.64–2.97) for NTIS in critically ill adults [11]. This literature supports considering low FT3 as an integrated biochemical marker of the response to illness. However, illness severity remains a competing explanation: in a selected ICU cohort, Zhong et al. found that the association of low total T3 with mortality weakened after adjustment for organ-failure severity [23]. The FT3 association in our study persisted after adjustment for demographic, inflammatory, and comorbidity covariates, but CRP and WBC do not replace direct measures of organ dysfunction, nutritional status, or frailty.

The interaction between FT4 and levothyroxine treatment deserves particular emphasis. Among nonusers, higher FT4 was associated with longer hospitalization, whereas the model estimates indicated approximately no association with hospitalization length in users. For in-hospital mortality, the FT4-by-treatment interaction was significant, and the point estimates suggested opposite directions of association between the groups. The nonuser mortality association did not reach conventional statistical significance, and a separate confidence interval for the within-user association is not presented. These findings indicate that the modeled FT4–outcome associations differed by treatment status; they do not establish that higher FT4 itself is harmful in nonusers or beneficial in users.

The changing physiology of NTIS provides a plausible context for this finding. FT4 is variable in NTIS and may be normal or elevated; with increasing illness severity and duration, circulating T4 can also fall [1,3,24]. Changes in peripheral hormone metabolism and binding can produce discordant FT3 and FT4 concentrations. Ataoğlu et al. found that combined low FT3 and elevated FT4 was associated with long-term mortality in hospitalized patients [24], supporting consideration of the hormone pattern rather than FT4 alone. In levothyroxine users, this pattern also reflects exogenous T4: athyreotic patients receiving LT4 can have higher FT4 and lower FT3 than controls despite normal TSH [5], a pattern also observed in our exposure groups. Dose, absorption, and residual thyroid function may further contribute. FT4 also varies with the interval between levothyroxine administration and blood sampling [25]. These factors are possible explanations for the treatment-specific associations, but their contributions were not measured in this cohort.

Measurement effects also warrant consideration. Free fatty acids can alter thyroid-hormone binding [26], and heparin-associated in vitro lipolysis can produce apparent FT4 elevation, particularly in equilibrium-dialysis experiments [27]. These are potential influences on inpatient thyroid testing, not evidence that our immunoassay measurements were affected. Medication-to-sampling timing and the contribution of such effects were not assessed.

The conditional associations with pre-admission levothyroxine use also deserve consideration. The IRR of 0.87 and OR of 0.53 compare users and nonusers at the same mean hormone concentrations, conditional on other model covariates. Users had lower mean FT3 and higher mean FT4 than nonusers, so these comparisons address a different question from comparison of the groups as observed. The similar crude hospital-stay and mortality outcomes illustrate this distinction. The conditional coefficients should therefore not be interpreted as population-average treatment benefits.

Continued exogenous T4 supply could influence hormone availability during illness, while changes in conversion and tissue signaling may persist despite replacement [1,3,10]. Whether pre-existing replacement modifies the physiological consequences of NTIS remains an untested biological hypothesis. Alternatively, medication adherence, absorption, and sustained outpatient care may reflect clinical stability and healthcare engagement. Longitudinal follow-up for hypothyroidism may be associated with differences in pre-admission medical care. These possibilities must be considered alongside confounding by the underlying thyroid disorder and acute illness. Conditioning on hormone concentrations that reflect both prior therapy and illness, and selecting patients who underwent hormone testing, may also introduce bias.

It is important to distinguish pre-admission levothyroxine use from initiating thyroid-hormone treatment during acute illness. The small trial by Brent and Hershman did not establish clinical benefit from T4 in severe non-thyroidal illness [19], while the brief T3 trial by Pingitore et al. in stable heart failure evaluated physiological outcomes [18]. Chu et al. examined levothyroxine administration within seven days after sepsis diagnosis in patients without documented hypothyroidism and observed higher mortality [28]. These exposure definitions and populations differ from ours, and neither observational study establishes a treatment effect. Replacement history and acute hormone administration therefore represent distinct research questions.

Several practical implications follow from our findings. FT4 should be interpreted alongside FT3, treatment history, the underlying thyroid diagnosis, and the acute clinical state. Low FT3 may have potential utility for risk stratification in selected hospitalized patients, but prospective studies must establish whether it adds information beyond clinical severity measures. The treatment-specific FT4 associations also justify prospective studies that characterize pre-illness thyroid status, dose, adherence, and sampling time, and investigate whether pre-existing replacement influences the response to acute illness. These research questions do not establish a dosing strategy. Management of established hypothyroidism remains distinct from initiating thyroid hormone specifically for NTIS; ATA guidance recommends against initiating LT4 or LT3 to treat NTIS in critically ill hospitalized adults [20].

Strengths include the very large underlying hospital population, matching on a broad set of measured covariates, formal treatment-by-hormone interaction analyses, and complete ascertainment of mortality. Several limitations qualify the findings. First, testing was selective: TSH ordering differed by department, and FT3/FT4 were discretionary throughout. Complete cases represented only 25.9% of the matched cohort, differed from excluded patients, and no longer retained the original matching balance. Hospital stay includes time before thyroid sampling, so prolonged illness may affect both the likelihood of testing and the hormone results. A single measurement and the absence of sampling-time and trajectory analyses limit temporal interpretation; death can also shorten hospitalization.

Second, treatment dose, duration, adherence, indication, and outpatient thyroid control were not characterized. TSH < 5 mIU/L permits suppressed TSH and does not identify a uniform NTIS or adequately replaced population. The broad thyroid exclusions, overlapping codes, and retained pituitary diagnoses in Table S1 limit diagnostic specificity. Restricted code sets may under-ascertain comorbidity, and diagnoses coded during admission may post-date exposure or acute illness.

Third, the covariate set did not include direct acute illness severity, frailty, functional status, nutritional measures, contemporaneous renal function, admission diagnosis, sepsis, malignancy, or relevant medications such as corticosteroids, amiodarone, beta-blockers, dopamine, and heparin. The disease indicators and inflammatory markers offer partial adjustment for these domains. Residual confounding by indication and severity therefore remains, and the conditional treatment coefficient should not be interpreted causally.

Fourth, the study spans changes in clinical care, electronic records, assay platforms, and the COVID-19 period, which may alter admission patterns, case mix, testing practices, and outcomes. FT3 availability confines the complete-case analysis to the later testing era and reduces, but does not document the elimination of, platform heterogeneity. Analyses stratified by department, assay, calendar period, thyroid diagnosis, or sampling time are not presented; temporal and subgroup consistency remain unestablished.

Finally, 179 mortality events supported 16 model terms excluding the intercept, including three interactions, and multiple nominal tests were conducted. Separate assessments of nonlinearity, calibration, influence, collinearity, and internal validation are not presented. Missing values were not imputed, and pair-adjusted uncertainty estimates are not reported. The event-to-term ratio alone is not evidence of model stability. The post-discharge analysis is unadjusted. The data came from a single general hospital, which further limits generalizability. Replication in other hospitals and prospective assessment are needed to establish the clinical utility of the FT4 interactions observed across the two inpatient outcomes.

## 5. Conclusions

In this cohort of tested medical inpatients, lower FT3 was associated with longer hospitalization and higher in-hospital mortality odds in nonusers, the reference group. Pre-admission levothyroxine use was associated with shorter hospitalization and lower mortality odds at mean thyroid-hormone concentrations. FT4–outcome associations differed by levothyroxine use for both inpatient outcomes. These findings support considering treatment history when interpreting thyroid tests and provide a rationale for prospective evaluation of their prognostic and physiological significance within the NTIS framework.

## Figures and Tables

**Table 1:** Characteristics of the propensity-score-matched parent cohort with TSH < 5 mIU/L (*n* = 14,332).

Characteristic	No Levothyroxine Treatment	Levothyroxine Treated	SMD
*n*	7166	7166	-
Age (years)	74.30 (12.94)	74.27 (12.89)	0.003
Female sex, proportion (SD)	0.78 (0.41)	0.78 (0.42)	0.007
Type 2/unspecified diabetes	0.12	0.12	0.014
Type 1 diabetes	0.01	0.01	0.005
Hypertension	0.53	0.53	0.012
Ischemic heart disease	0.22	0.22	0.004
Hyperlipidemia	0.30	0.30	0.007
Heart failure	0.15	0.16	0.031
Non-alcoholic liver disease	0.01	0.01	0.034
Renal disease	0.06	0.06	0.023
Chronic liver disease/cirrhosis	0.01	0.01	0.014
Anemia	0.10	0.11	0.025
Obesity/overweight	0.13	0.14	0.018
Chronic respiratory failure	<0.01	<0.01	0.016
Chronic obstructive asthma	<0.01	<0.01	<0.001
Obstructive sleep apnea	0.01	0.01	0.017
Cerebral infarction	0.01	0.01	0.023
Other chronic conditions	0.15	0.15	<0.001

Age is shown as mean (SD); binary indicators are shown as proportions, with SD where tabulated. SMD = standardized mean difference. These balance measures concern the matched parent cohort. Disease indicators use the restricted code definitions in Table S1; the labels do not imply comprehensive clinical ascertainment of every condition. CKD = chronic kidney disease; the renal indicator also includes unspecified renal failure.

**Table 2:** Characteristics and unadjusted outcomes of the complete-case cohort (*n* = 3717).

Variable	No Levothyroxine (*n* = 1731)	Levothyroxine Use (*n* = 1986)	*p*-Value	Absolute SMD
Age (years)	75.20 ± 12.69	74.72 ± 13.11	0.35	0.037
Female sex, *n* (%)	1302 (75.2)	1466 (73.8)	0.33	0.032
TSH mIU/L	1.82 ± 1.15	1.86 ± 1.41	0.36	0.031
FT3 pmol/L	3.20 ± 1.07	2.73 ± 1.01	<0.001	0.452
FT4 pmol/L	15.70 ± 3.72	17.19 ± 5.38	<0.001	0.322
CRP mg/L	38.71 ± 70.93	46.22 ± 79.33	<0.001	0.100
Heart failure, *n* (%)	176 (10.2)	263 (13.2)	0.004	0.096
Renal disease indicator, *n* (%)	216 (12.5)	302 (15.2)	0.02	0.079
Length of hospitalization (days)	7.71 ± 13.09	7.57 ± 15.06	0.99	—
In-hospital mortality, *n* (%)	87 (5.0)	92 (4.6)	0.58	—
Death within 31 days after discharge, *n*/*N* (%)	63/1644 (3.8)	65/1894 (3.4)	0.525	—

Values are mean ± SD or *n* (%), except post-discharge mortality, shown as *n*/*N* (%). Its denominators exclude the 87 nonusers and 92 users who died in hospital: 1644 and 1894 survivors, respectively. Mortality follow-up was complete. Continuous-variable SMDs are approximate because they use rounded means and SDs; binary SMDs use exact counts. SMDs describe characteristics, not outcomes. The post-discharge *p*-value is an unadjusted Pearson χ^2^ test. CRP = C-reactive protein; FT3 = free triiodothyronine; FT4 = free thyroxine; TSH = thyroid-stimulating hormone.

**Table 3:** Multivariable negative binomial regression for length of hospitalization (*n* = 3717).

Variable	IRR	95% CI	*p*
Female sex	0.96	0.88–1.04	0.278
Age (years)	1.004	1.001–1.007	0.006
Levothyroxine treatment	0.87	0.81–0.94	<0.001
TSH mIU/L	1.00	0.95–1.04	0.880
FT3 pmol/L	0.76	0.73–0.80	<0.001
FT4 pmol/L	1.05	1.03–1.06	<0.001
WBC 10^9^/L	1.01	1.00–1.02	0.002
CRP mg/L	1.00	1.00–1.00	0.077
Type 2/unspecified diabetes	0.94	0.85–1.04	0.196
Hypertension	1.11	1.02–1.21	0.013
Ischemic heart disease	1.06	0.95–1.19	0.306
Heart failure	1.17	1.05–1.30	0.006
Renal disease	1.05	0.94–1.17	0.383
TSH × levothyroxine	0.99	0.94–1.05	0.821
FT3 × levothyroxine	1.10	1.03–1.18	0.005
FT4 × levothyroxine	0.95	0.94–0.97	<0.001

Values are IRRs with 95% CIs from a negative binomial model with log link. For this outcome, the IRR is a ratio of expected hospital days. Continuous covariates were mean-centered. Hormone main terms refer to nonusers; the levothyroxine term refers to the mean-centered hormone reference values. Interaction terms are ratios of the user and nonuser hormone IRRs, rather than the within-user IRRs themselves. The model includes all 16 terms shown.

**Table 4:** Multivariable logistic regression for in-hospital mortality (*n* = 3717; mortality events = 179 [4.8%]).

Variable	OR	95% CI	*p*
Female sex	0.91	0.64–1.30	0.610
Age (years)	1.06	1.04–1.08	<0.001
Levothyroxine treatment	0.53	0.35–0.82	0.004
TSH mIU/L	0.99	0.81–1.20	0.916
FT3 pmol/L	0.47	0.35–0.62	<0.001
FT4 pmol/L	1.06	1.00–1.13	0.057
WBC 10^9^/L	1.01	1.00–1.03	0.038
CRP mg/L	1.003	1.001–1.004	<0.001
Type 2/unspecified diabetes	1.15	0.75–1.76	0.518
Hypertension	0.90	0.61–1.31	0.581
Ischemic heart disease	1.60	1.03–2.49	0.038
Heart failure	1.14	0.74–1.77	0.556
Renal disease	1.08	0.72–1.62	0.715
TSH × levothyroxine	0.90	0.70–1.16	0.408
FT3 × levothyroxine	1.06	0.71–1.58	0.783
FT4 × levothyroxine	0.83	0.77–0.90	<0.001

Values are ORs with 95% CIs from logistic regression with the 16 terms shown. Continuous covariates were mean-centered. Hormone main terms refer to nonusers and the levothyroxine term to the mean-centered hormone reference values. Interaction ORs compare the per-unit hormone associations between groups. ORs describe odds, not risk ratios; confidence intervals for combined within-user associations require the coefficient variances and covariance.

## Data Availability

The de-identified data presented in this study are available from the corresponding author upon reasonable request and subject to approval by Bnai Zion Medical Center.

## Supplementary Materials

The following supporting information can be downloaded at https://www.mdpi.com/article/10.3390/jcm15218382/s1: Table S1: ICD-9-CM codes used to define excluded thyroid diseases and comorbidities used for the propensity score; Table S2: Comparison of baseline characteristics between the complete-case sample and patients excluded because of missing data.
