# Supplementary material for: Beyond Euthyroid Sick Syndrome: Pre-Admission Levothyroxine Use and Thyroid Hormone–Outcome Associations in Hospitalized Patients
Source: J Clin Med. 2026 Oct 28;15(21):8382. doi: 10.3390/jcm15218382 (PMC13763610; doi:10.3390/jcm15218382)
Supplement: Supplementary file 1 [file jcm-15-08382-s001.zip › jcm-4577441-supplementary.pdf]

Beyond Euthyroid Sick Syndrome: Pre-admission Levothyroxine Use and Thyroid Hormone–Outcome Associations in Hospitalized Patients

Supplementary Materials

Mohammad Sheikh-Ahmad et al.

Table S1. ICD-9-CM code definitions for thyroid-disease exclusions and comorbidity indicators.

Background/admission thyroid diseases excluded

**Condition/diagnosis**	**ICD-9-CM codes**
Thyrotoxicosis	242.X
Toxic Diffuse Goiter	242.0
Toxic Diffuse Goiter with thyrotoxic crisis/storm	242.01
Toxic Uninodular Goiter	242.1
Toxic Uninodular Goiter with thyrotoxic crisis/storm	242.11
Toxic Multinodular Goiter	242.2
Toxic Multinodular Goiter with thyrotoxic crisis/storm	242.21
Toxic nodular goiter, unspecified type	242.3
Thyrotoxicosis of other specified origin	242.8
Thyrotoxicosis of other specified origin with thyrotoxic crisis/storm	242.81
Thyrotoxicosis without mention of goiter or other cause	242.9
Thyrotoxicosis without mention of goiter or other cause, with Thyrotoxic crisis/storm	242.91
Thyroiditis	245.X
Acute Thyroiditis	245.0
Subacute Thyroiditis	245.1
Iatrogenic Thyroiditis	245.4
Other and unspecified chronic thyroiditis	245.8
Thyroiditis, unspecified	245.9
Other specified disorders of thyroid	246.8

Comorbidity indicators coded before or during admission

**Condition/diagnosis**	**ICD-9-CM codes**
Type 2 or unspecified diabetes (without mention of complications)	250.00, 250.02
Type 1 diabetes (without mention of complications)	250.01, 250.03
Hypertension	401.10, 401.90
Selected chronic ischemic heart disease / old myocardial infarction codes	414.90, 412.00
Hyperlipidemia	272.20, 272.40
Heart failure	428.22, 428.32, 428.42, 428.00
Chronic nonalcoholic or unspecified liver disease	571.80, 571.90
Chronic kidney disease or unspecified renal failure	585.60, 585.90, 586.00
Chronic nonalcoholic liver disease / cirrhosis	571.80, 571.50
Anemia	285.90, 285.29, 285.21
Obesity or overweight	278.00, 278.01, 278.02
Chronic respiratory failure	518.83
Chronic obstructive asthma (COPD indicator)	493.20
Obstructive sleep apnea	327.23, 780.57
Cerebral arterial occlusion with infarction	434.91
**Other chronic conditions**	
Depression	311.00
Osteoporosis	733.00
Amyloidosis	277.30
Alzheimer disease	331.00

Condition/diagnosis	ICD-9-CM codes
Anxiety disorder	300.00, 293.84
Adrenal neoplasms / other adrenal disorders	227.00, 237.20, 255.80, 255.90
Nodular goiter code grouping	241.00, 241.10, 241.90, 242.30
Cushing syndrome	255.00
Panhypopituitarism	253.20
Acromegaly	253.00
Hyperparathyroidism	252.80, 252.01, 252.00, 252.02

Covariate structure: cerebrovascular disease was a separate indicator, and diagnoses under "Other chronic conditions" formed one composite indicator. Atrial fibrillation was not a separate propensity-score covariate. Codes were assigned before or during admission. The detailed code sets define the analytic indicators and may capture narrower populations than broad clinical labels suggest.

The code families overlap: 242.30 lies within the excluded 242.X family, and 571.80 appears in both liver-disease indicators. The listed 245.X thyroiditis family includes chronic lymphocytic thyroiditis, while the composite includes pituitary diagnoses. These features limit interpretation of thyroid-disease etiology and comorbidity prevalence; the indicators should not be read as an adjudicated diagnosis of NTIS or as proof that all central thyroid dysfunction was excluded.

Table S2. Comparison of baseline characteristics between the complete-case sample and patients excluded because of missing data.

Characteristic	Excluded patients	Complete-case sample	Effect size
n	10,615	3,717	-
Age (years), mean (SD)	74.05 (12.91)	74.95 (12.92)	0.07
Female sex	0.79	0.75	0.05
Type 2/unspecified diabetes	0.10	0.17	0.10
Type 1 diabetes	0.01	0.01	0.03
Hypertension	0.57	0.41	0.14
Ischemic heart disease (coded)	0.26	0.12	0.15
Hyperlipidemia	0.31	0.27	0.04
Heart failure	0.17	0.12	0.06
Non-alcoholic liver disease	0.01	0.01	0.01
Renal disease (coded)	0.03	0.14	0.20
Chronic liver disease/cirrhosis	0.01	0.01	0.04
Anemia	0.09	0.13	0.06
Obesity/overweight	0.15	0.08	0.09
Chronic respiratory failure	< 0.01	< 0.01	0.03
Chronic obstructive asthma	< 0.01	< 0.01	0.03
Obstructive sleep apnea	0.01	0.01	0.01
Cerebral infarction (coded)	< 0.01	0.03	0.11
Other chronic conditions	0.16	0.12	0.04

Age is shown as mean (SD); binary variables are proportions. Effect sizes are Cohen's d or Cramér's V and describe complete-case selection, rather than balance between levothyroxine users and nonusers. Differences in hypertension, ischemic heart disease, and renal disease demonstrate that similar age and sex distributions do not make the two samples interchangeable.
